# An economic evaluation of cabazitaxel versus a second androgen receptor-targeted agent (ARTA) for patients with metastatic castration-resistant prostate cancer previously treated with docetaxel and an ARTA: the United States payer perspective

**DOI:** 10.1186/s12913-022-08274-x

**Published:** 2022-07-14

**Authors:** Alicia K. Morgans, Thomas Hutson, Alice Kai Dan Guan, David Garcia, Anna Zhou, Edward Drea, Nicholas J. Vogelzang

**Affiliations:** 1 Dana-Farber Cancer Institute, 450 Brookline Avenue, Boston, MA 02215 USA; 2 US Oncology, Texas Oncology, Dallas, TX USA; 3 CRG-EVERSANA Canada Inc., 3228 South Service Road, Suite 204, Burlington, ON L7N 3H8 Canada; 4 Sanofi, Cambridge, MA USA; 5 US Oncology Research, Las Vegas, NV USA

**Keywords:** Cabazitaxel, Androgen receptor-targeted agent, Economic impact, Metastatic castration-resistant prostate cancer, Third-line treatment, Symptomatic skeletal events, End-of-life care

## Abstract

**Background:**

Cabazitaxel significantly improves clinical outcomes compared with a second androgen receptor-targeted agent (ARTA) in patients with metastatic castration-resistant prostate cancer (mCRPC) previously treated with docetaxel and an ARTA (abiraterone or enzalutamide), as demonstrated in the CARD trial (NCT02485691). We aimed to estimate healthcare costs avoided with the use of cabazitaxel as a third-line (3 L) treatment versus a second ARTA from a US payer perspective.

**Methods:**

Model inputs were based on the CARD trial, published sources, and estimates of typical clinical care patterns by genitourinary oncologists (*n* = 3). Assessed time points were 6, 12, 18, and 24 months. Outcomes included progression-free survival (PFS), radiographic PFS (rPFS), and overall survival (OS); hospitalization and intensive care unit (ICU) days; and costs (reported in 2020 US dollar [USD] and converted into Euro) to manage symptomatic skeletal events (SSEs), adverse events (AEs), and end-of-life care.

**Results:**

At 18 months, in a cohort of 100 patients, the use of cabazitaxel was estimated to result in 9 more patients achieving rPFS, 2 more patients achieving PFS, and 17 more survivors versus a second ARTA. The costs of SSEs, AEs, and end-of-life care were $498,909 (€424,073), $276,198 (€234,768), and $808,785 (€687,468), respectively, for cabazitaxel and $627,569 (€533,434), $251,124 (€213,455), and $1,028,294 (€874,050), respectively, for a second ARTA. Cabazitaxel was estimated to be associated with a 21% reduction in both SSE management and end-of-life care costs. Hospitalization cost was $1,442,870 (€1,226,440) for cabazitaxel and $1,728,394 (€1,469,135) for a second ARTA, representing an estimated 17% reduction in these costs. Cabazitaxel, as compared with a second ARTA, was associated with 58 fewer hospitalization days and 2 fewer ICU days and was estimated to avoid $323,095 (€274,630, 17%) in total costs, driven by SSEs management and end-of-life care.

**Conclusion:**

The use of cabazitaxel as a 3 L treatment after docetaxel and an ARTA in patients with mCRPC is estimated to result in clinical benefits (longer rPFS, PFS, and OS) and lower healthcare resource utilization (fewer hospitalization and ICU days), compared with a second ARTA.

**Supplementary Information:**

The online version contains supplementary material available at 10.1186/s12913-022-08274-x.

## Background

Prostate cancer is the second leading cause of cancer-related mortality among men in the United States (US) [1]. In 2020, 191,930 new cases of prostate cancer were diagnosed in the US; about 10 to 20% of these cases are likely to develop into castration-resistant prostate cancer (CRPC) within 5 years of diagnosis [1]. Approximately 41% of patients with CRPC are metastatic at diagnosis [2]. Several therapies, including taxanes (e.g., cabazitaxel and docetaxel), androgen receptor-targeted agent (ARTA or androgen-signaling-targeted inhibitor [ASTI]; e.g., enzalutamide and abiraterone), a radiopharmaceutical agent (e.g., radium-223 and Lutetium-177), and an immunotherapy agent (e.g., sipuleucel-T), have improved survival for patients with metastatic CRPC (mCRPC) [3–9]. Docetaxel is the recommended first-line chemotherapy in patients with mCRPC [10]. In addition to docetaxel, ARTAs are commonly administered in the earlier stages of mCRPC [11].

After receiving docetaxel, patients who progress while receiving an ARTA may have a marginal response when switched to an alternative ARTA [12–17]. In contrast, studies have suggested that cabazitaxel retains a high level of anti-tumor activity in patients who have had disease progression while receiving docetaxel or ARTAs [3, 18]. In the CARD trial, cabazitaxel demonstrated a significant improvement in clinical outcomes including radiographic progression-free survival (rPFS, *p* < 0.001), PFS (*p* < 0.001), and overall survival (OS, *p* = 0.008) compared with a second ARTA [19]. Despite the favourable outcomes from the CARD trial, an optimal cost-effective third-line (3 L) treatment for managing patients with mCRPC previously treated with docetaxel and an ARTA is currently unclear. Additionally, even though the current treatments have improved the median OS in patients with mCRPC [20], they are associated with higher healthcare resource utilization (HCRU), including for the management of symptomatic skeletal events (SSEs) and adverse events (AEs) [21]. McDougall et al. (2016) studied the economic impact of SSEs among Medicare-enrolled men with metastatic prostate cancer and determined that the attributable cost of ≥1 SSE management was $21,191 (US dollar [USD] 2016) [22]. A recent systematic review of cost-effectiveness and cost analyses reported that the annual direct healthcare cost of patients with mCRPC ranged between $26,707 and $67,957 (USD 2015) [23].

In countries like the US, the economic aspect has a greater influence on the clinician’s therapeutic choice than in other healthcare contexts [24]. Healthcare decision makers face a significant challenge to optimize the treatment landscape in terms of cost and effectiveness in patients with mCRPC previously treated with docetaxel who had progressed within 12 months while receiving an ARTA. To address this unmet need, based on the results of the CARD study, we developed an economic model to quantify the clinical outcomes, including rPFS, PFS, OS, hospitalization days, and intensive care unit (ICU) days, and to determine potential HCRU and associated costs avoided, from the United States payers’ perspective, in a hypothetical cohort of patients with mCRPC receiving cabazitaxel as a 3 L treatment compared with a same size cohort receiving a second ARTA.

## Methods

### Analysis overview

An economic model was developed in Microsoft Excel to compare cabazitaxel as a 3 L treatment with a second ARTA (abiraterone or enzalutamide) in patients with mCRPC from the US payers’ and population healthcare decision makers’ perspective. Efficacy and safety inputs were based on results from the CARD trial [19]. Inputs from three genitourinary oncologists (Alicia Morgans, Thomas Hutson, and Nicholas J. Vogelzang) were used to validate the assumptions and to inform clinical parameters, such as routine treatment of AEs, expected rates of hospitalization, and length of stay (LOS) for Grade 3/4 AEs and SSEs, which were not available in the published literature.

Given the short life expectancy for the target population (median OS of 11.0–13.6 months) [19], a time horizon of 18 months was used for the reference case analysis, and scenario analyses were conducted for time horizons of 6, 12, and 24 months. The clinical outcomes assessed at the various time points (6, 12, 18, and 24 months) included rPFS, PFS, OS, and hospitalization days. The economic analysis included the costs associated with the management of SSEs, AEs, and end-of-life care. Costs specifically associated with inpatient stays were also estimated. All costs were adjusted to 2020 US dollars (USD, translated in Euro (€) to suit local readers, 1 US dollar equals 0.85 Euro per exchange rate as of August 30, 2021). Only direct costs were considered as the analysis was conducted from the US payers’ and population healthcare decision makers’ perspective.

### Clinical inputs

This analysis estimated the proportion of patients achieving rPFS, PFS, and OS at 6, 12, 18, and 24 months using the Kaplan–Meier (KM) curves of respective events from the CARD trial (Supplementary Appendix 1). The incidence of SSEs (i.e., pathological fracture, radiation to bone, spinal cord compression, surgery to bone) was calculated as the overall monthly rate of SSEs multiplied by the total months of OS at 6, 12, 18, and 24 months for each treatment. The distribution of type of SSE was obtained from the CARD trial [25]. The overall monthly rates of SSEs were estimated based on the treatment-specific probabilities of SSEs and mean follow-up time reported from the CARD trial and the formula to convert probabilities to rates described by Fleurence and Hollenbeak (2007) [26] (Supplementary Appendix 2). To estimate the total months of OS at various time points, the OS KM curve was digitized (using Digitizelt software, version 2.5.3), and the median time of survival for patients with events during the intervals of interests (i.e., 0–6, 6–12, 12–18, and 18–24 months) was estimated. Then, the total time of survival at each time point was calculated. Supplementary Appendix 3 presents the estimates of total months of OS and number of SSEs for a cohort of 100 patients at each time point.

This analysis considered all treatment-related Grade 3/4 AEs based on the National Cancer Institute (NCI) Common Terminology Criteria for Adverse Events (CTCAE) (National Cancer Institute, 2009), including laboratory abnormalities that occurred in ≥3% of patients, in either treatment arm, in the CARD trial. Based on clinician input, events for which there is no routine treatment (i.e., an increase in aspartate transaminase or alanine transaminase) were excluded. The incidence of AEs included in the analysis is summarized in Supplementary Appendix 4. To estimate the total number of AEs, the incidence of events was multiplied by the hypothetical cohort size (*n* = 100). The proportions of patients with SSEs or Grade 3/4 AEs or in end-of-life care who are expected to be hospitalized or placed in the ICU, as well as the number of days in the hospital or ICU for SSEs management and Grade 3/4 AEs, were based on clinician input (Supplementary Appendix 5). The distribution of type of SSE was applied to estimate the total number of SSEs at 6, 12, 18, and 24 months (Supplementary Appendix 6). The number of hospitalization days for end-of-life care was obtained from Wilson et al. (2014) [27]. The rates of hospitalization and ICU admission and the LOS for each event were assumed to be the same for both cabazitaxel and a second ARTA. We excluded events for which hospital/ICU admission was not routinely expected (e.g., radiation to bone, musculoskeletal pain/discomfort, peripheral neuropathy, etc.).

### Healthcare resource use costs

United States-specific estimates of HCRU costs were used in this model and were obtained from published literature [27–32]. Costs were analyzed in 2020 US dollars; in the instances where the costs were available only from previous years, the costs were inflated using the health component of the Consumer Price Index [33]. Table 1 presents the costs of SSE management and costs of Grade 3/4 AE management with their sources used in this analysis.

**Table 1:** Published US costs of management of symptomatic skeletal events and Grade 3/4 adverse events

Event	Cost	Source of cost^a^
Symptomatic skeletal events^b^		
Radiation to bone	$6460 (€5491)	Carter et al. (2013) [30]
Pathological fracture	$31,387 (€26,679)	Carter et al. (2013) [30]
Spinal cord compression	$46,382 (€39,425)	Carter et al. (2013) [30]
Grade 3/4 adverse events		
Asthenia or fatigue	$27 (€23)	Sorensen et al. (2013) [32]
Diarrhea	$8268 (€7028)	Bui et al. (2016 ) [29]^c^
Infection	$9689 (€8236)	Bui et al. (2016 ) [29]^c^
Musculoskeletal pain or discomfort	$19 (€16)	Sorensen et al. (2013) [32]
Peripheral neuropathy	$748 (€636)	Costing methodology: Bilir et al. (2016 ) [28]^d^
Renal disorder	$11,713 (€9956)	Bui et al. (2016) [29]^c^
Cardiac disorder	$13,126 (€11,157)	Bui et al. (2016 ) [29]^c^
Febrile neutropenia	$18,739 (€15,928)	Bui et al. (2016 ) [29]^c^
Anemia	$5063 (€4304)	Sorensen et al. (2013) [32]
Leukopenia	$191 (€162)	Roy et al. (2015) [31]
Neutropenia	$191 (€162)	Roy et al. (2015) [31]
Thrombocytopenia	$1266 (€1076)	Sorensen et al. (2013) [32]
Hyponatremia^e^	$1354 (€1151)	Cost of outpatient management: Roy et al. (2015) [31] Cost of inpatient management: Bilir et al. (2016) [28]

*Abbreviations*: *CPT* Current Procedural Terminology; *LOS* length of stay, *US* United States, *USD* US dollar

^a^Reported costs were inflated to 2020 USD using the health component of the Consumer Price Index [33]

^b^The cost for bone surgery was not included as the incidence was 0% for both arms in the CARD trial [25]

^c^Costs reported by Bui et al. [29] assumed hospitalization (aligned with clinician input)

^d^Cost based on CPT 99214 (outpatient visit, $110.43 [€94]) [42] and Red Book [43] cost for pregabalin ($11.19 [€10]). Pregabalin dosage: 300 mg/day for 3 days + 600 mg/day for 27 days

^e^Assumed 92.5% outpatient management and 7.5% hospitalization with 3 days of LOS (based on clinician input)

The cumulative costs for AEs at the four time points of interest (i.e., 6, 12, 18, and 24 months) were based on the cumulative proportion of treatment administered by each time point during the CARD trial. A summary of the cumulative proportion of treatment administered and AE-related costs at each time point is provided in Supplementary Appendix 7.

Based on clinician input, it was assumed that 10% of total deaths would happen following hospitalization. The cost of end-of-life care for patients who died during hospitalization was estimated to be $130,660 (€111,061) based on Wilson et al. (2014) [27]. This cost included the average cost of the last hospitalization for severe side effects (neutropenia and cardiac events) for an average stay of 22 days.

The cost per hospitalization day (assuming the inclusion of ICU costs) due to Grade 3/4 AEs and SSEs was calculated for each event as follows:

- For Grade 3/4 AEs with a 100% rate of hospitalization (i.e., diarrhea, infection, renal disorder, cardiac disorder, and febrile neutropenia), the cost per event was divided by the number of expected days of hospitalization per event.
- For AEs with less than 100% rate of hospitalization (i.e., anemia, thrombocytopenia, and hyponatremia), the cost per day was obtained from Bilir et al. (2016) [28], which provided data on both mean inpatient cost and mean LOS for these events.
- For pathological fracture and spinal cord compression, published costs for these events were divided by the expected days of hospitalization (assuming that the costs were reflective of hospitalization costs).

Finally, to calculate the total cost of hospitalization, the estimated cost per day for each event (Table 2) were multiplied by the respective number of hospitalization days for each event, and the costs of overall events were summed for 6, 12, 18, and 24 months.

**Table 2:** US cost per hospitalization day for Grade 3/4 adverse events, symptomatic skeletal events, and end-of-life care

Event	US cost per hospitalization day
Diarrhea	$4134 (€3514)^a^
Infection	$2422 (€2059)^a^
Renal disorder	$2928 (€2489)^a^
Cardiac disorder	$3282 (€2790)^a^
Febrile neutropenia	$4685 (€3982)^a^
Anemia	$6111 (€5194)^b^
Thrombocytopenia	$5099 (€4334)^b^
Hyponatremia	$5232 (€4447)^b^
Pathological fracture	$6277 (€5335)^a^
Spinal cord compression	$9276 (€7885)^a^
End of life	$5939 (€5048)^c^

*Abbreviations*: *ICU* intensive care unit, *US* United States

ICU costs were assumed to be part of the hospitalization costs

^a^To obtain the respective cost, hospitalization costs per event were divided by the length of stay or expected days of hospitalization (based on clinician input) (Supplementary Appendix 5)

^b^Adapted from Bilir et al. (2016) [28]

^c^Adapted from Wilson et al. (2014) [27]

## Results

### Reference case analysis at 18 months

#### Number of patients achieving rPFS, PFS, and OS at 18 months

**Fig. 1:**
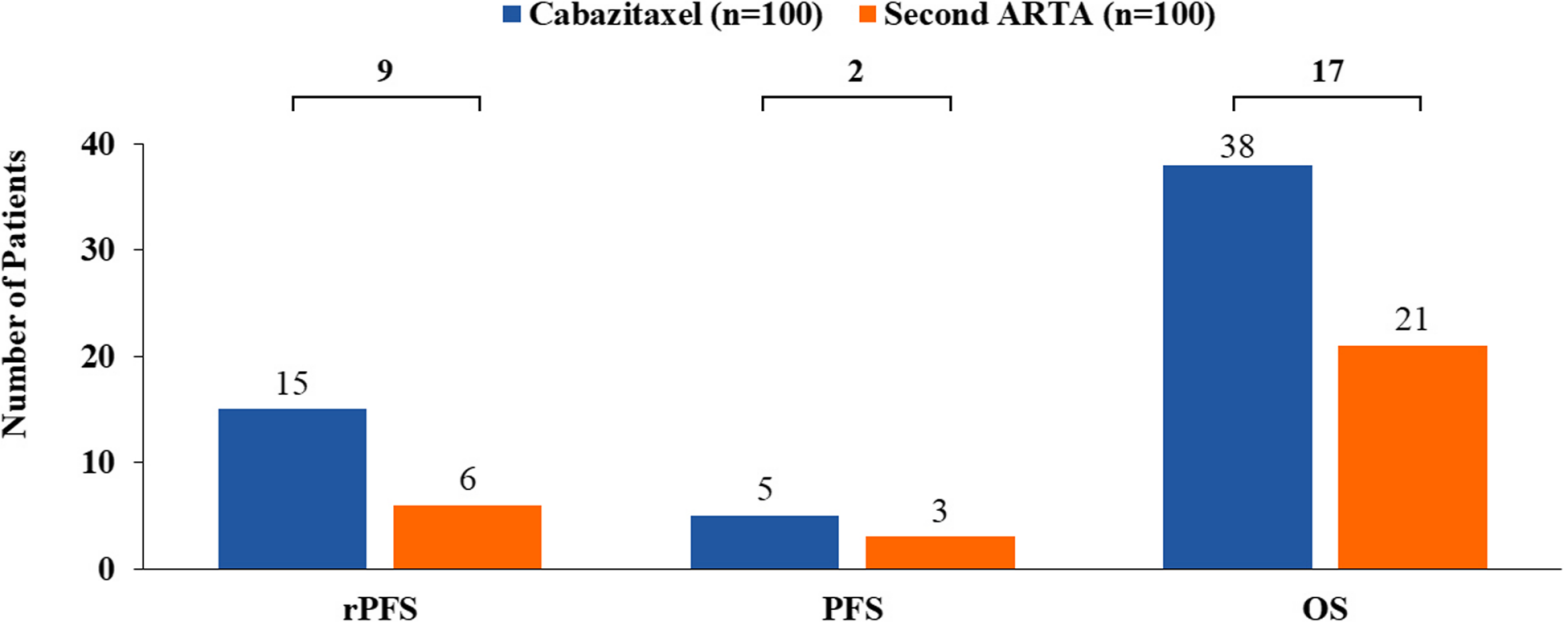
Number of patients in rPFS, PFS, and OS at 18 months. ARTA, androgen receptor-targeted agent; OS, overall survival; PFS, progression-free survival; rPFS, radiographic progression-free survival. The number correspond to a cohort of 100 patients for each treatment

For a cohort of 100 patients with mCRPC, the use of cabazitaxel as a 3 L treatment was estimated to result in 9 more patients achieving rPFS, 2 more patients achieving PFS, and 17 more survivors at 18 months compared with a similar cohort of patients receiving a second ARTA (Fig. 1).

#### Hospitalization and intensive care unit days at 18 months

**Fig. 2:**
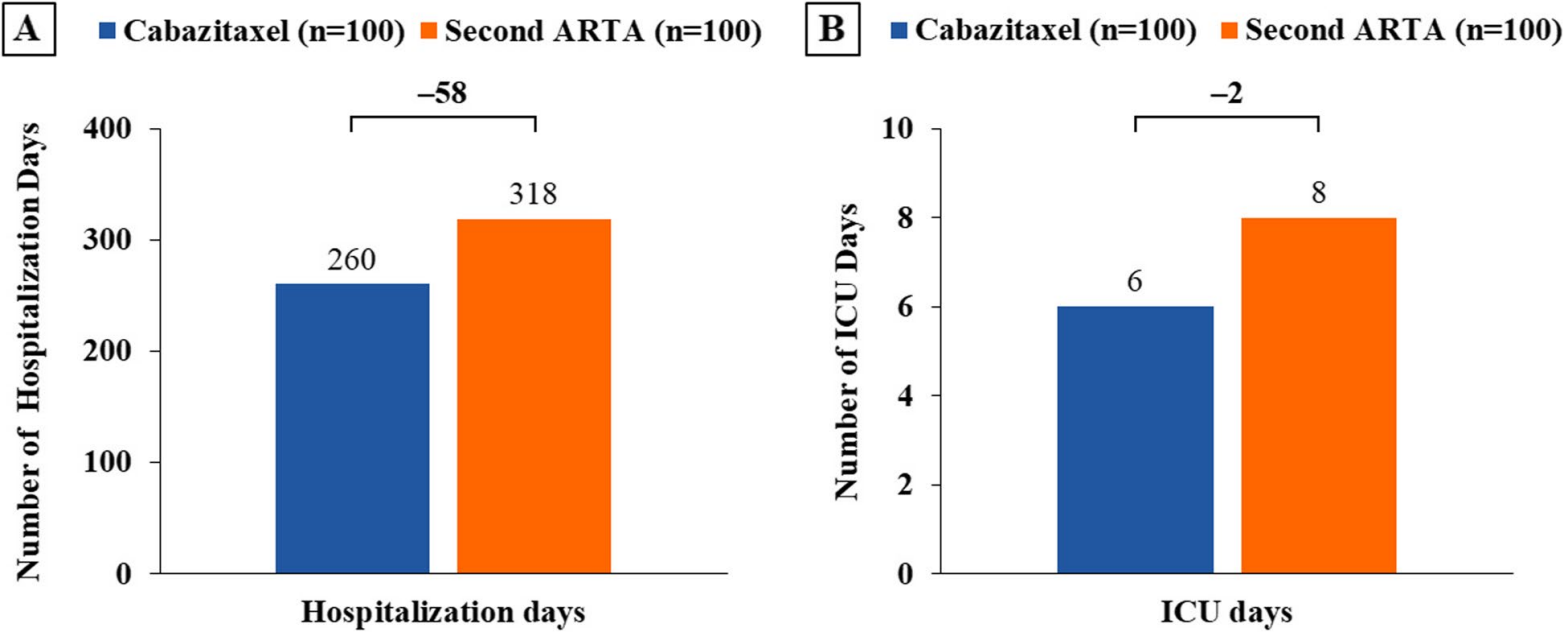
Hospitalization days (**A**) and ICU days (**B**) at 18 months. ARTA, androgen receptor-targeted agent; ICU, intensive care unit. The number correspond to a cohort of 100 patients for each treatment

At 18 months, the use of cabazitaxel as a 3 L treatment in patients with mCRPC was estimated to result in 58 fewer hospitalization days (Fig. 2A) and 2 fewer ICU days (Fig. 2B) than in a similar cohort of patients receiving a second ARTA.

#### Healthcare resource utilization and hospitalization costs

**Fig. 3:**
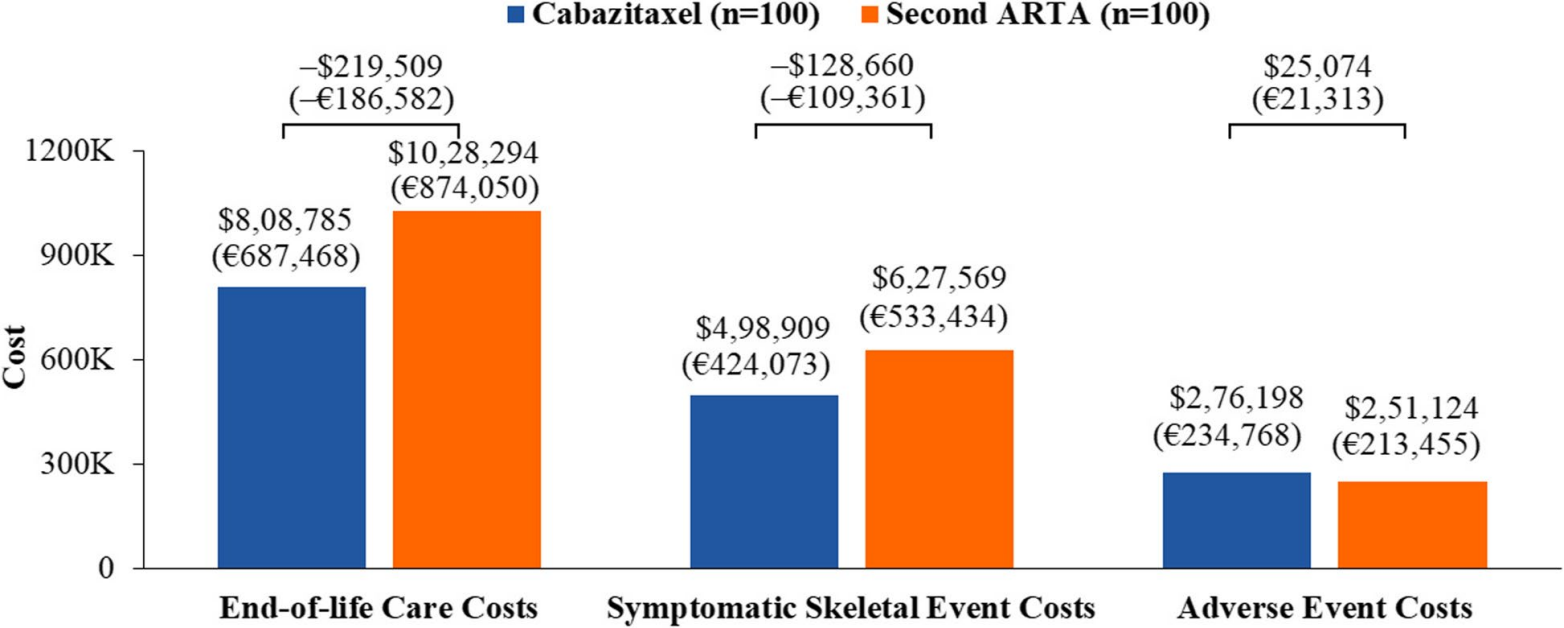
Healthcare resource utilization costs at 18 months. ARTA, androgen receptor-targeted agent. The number correspond to a cohort of 100 patients for each treatment

In patients with mCRPC, the use of cabazitaxel as a 3 L treatment was estimated to avoid $323,095 (€274,630) in additional HCRU costs at 18 months compared with patients receiving a second ARTA. These HCRU cost savings were due to decreased costs of SSE management and end-of-life care (Fig. 3).

**Fig. 4:**
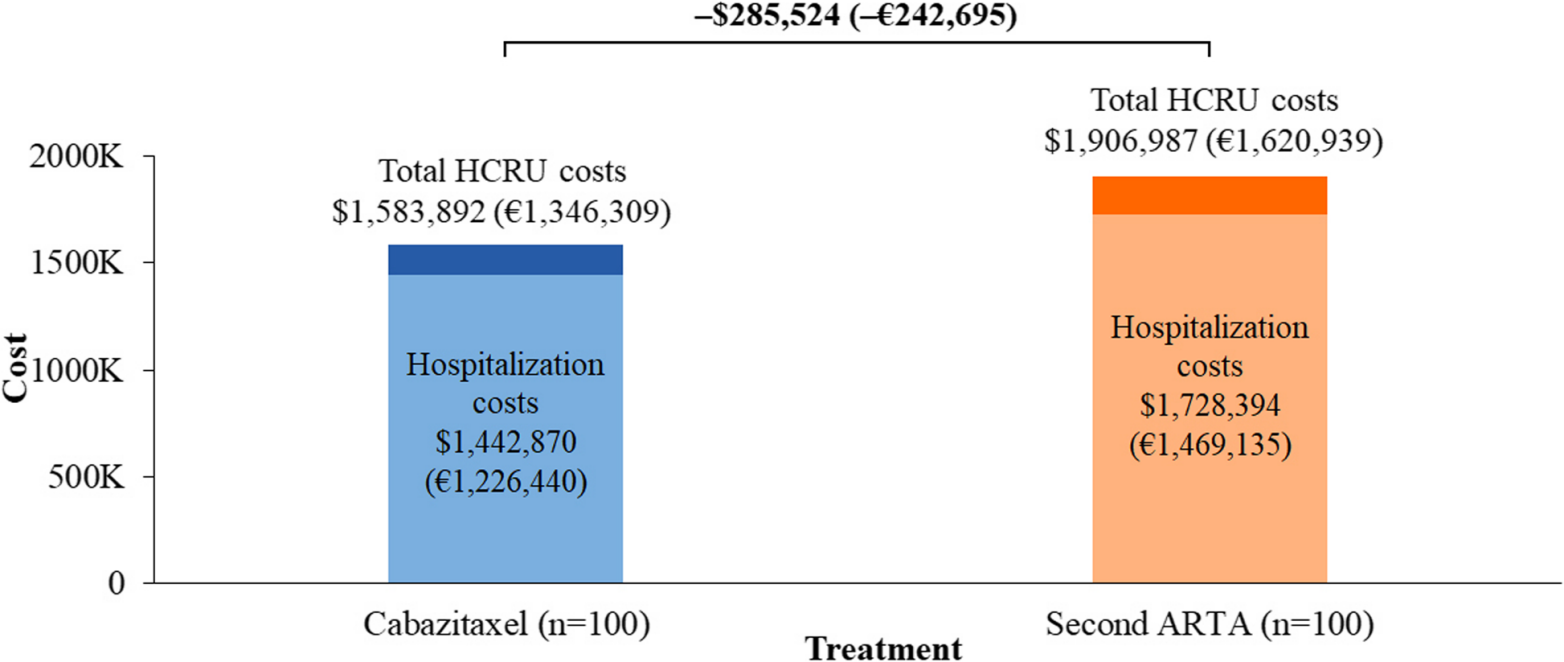
Hospitalization and overall HCRU costs at 18 months. ARTA, androgen receptor-targeted agent; HCRU, healthcare resource utilization. The number correspond to a cohort of 100 patients for each treatment

Hospitalization costs were estimated to constitute approximately 90% of the total HCRU costs for both cabazitaxel and a second ARTA (Fig. 4). At 18 months, hospitalization cost for cabazitaxel as a 3 L treatment was estimated as $1,442,870 (€1,226,440), whereas for a second ARTA, it was $1,728,394 (€1,469,135). Thus, cabazitaxel as a 3 L treatment was associated with a savings of $285,524 (€242,695) in hospitalization-related costs, compared with a second ARTA (Fig. 4). See Supplementary Appendix 8 for a summary of hospitalization-related costs.

### Scenario analyses at 6, 12, and 24 months

Clinical and cost results at 6, 12, and 24 months were consistent with the reference case results at 18 months (Tables 3 and 4).

**Table 3:** Number of patients in rPFS, PFS, and OS at 6, 12, and 24 months

Outcome	6 months			12 months			24 months		
	Cabazitaxel	Second ARTA	Difference	Cabazitaxel	Second ARTA	Difference	Cabazitaxel	Second ARTA	Difference
rPFS (number of patients)	58	36	22	27	9	18	6	4	2
PFS, (number of patients)^a^	36	16	21	10	3	7	0	0	0
OS, (number of patients)	86	81	5	56	45	12	25	9	16
Hospitalization days	112	138	−26	206	250	−44	297	351	−54
ICU days	5	7	−2	6	8	−2	7	8	−1

*Abbreviations*: *ARTA* androgen receptor-targeted agent, *ICU* intensive care unit, *OS* overall survival, *PFS* progression-free survival, *rPFS* radiographic progression free survival

Values correspond to a cohort of 100 patients for each treatment

^a^No results available for 24 months as the number at risk for PFS was 0 for both arms in the CARD trial

**Table 4:** Healthcare resource utilization costs at 6, 12, and 24 months

Outcome	6 months			12 months			24 months		
	Cabazitaxel	Second ARTA	Difference	Cabazitaxel	Second ARTA	Difference	Cabazitaxel	Second ARTA	Difference
SSEs	$219,191 (€186,312)	$309,285 (€262,892)	−$90,094 (−€76,580)	$394,870 (€335,640)	$519,906 (€441,920)	−$125,036 (−€106,281)	$566,887 (€481,854)	$674,083 (€572,971)	−$107,196 (−€91,117)
AEs	$226,750 (€192,738)	$202,843 (€172,417)	$23,907 (€20,321)	$267,456 (€227,338)	$234,821 (€199,598)	$32,635 (€27,740)	$277,018 (€235,465)	$256,140 (€217,719)	$20,878 (€17,746)
End-of-life care	$181,617 (€154,374)	$248,254 (€211,016)	−$66,637 (−€56,641)	$569,678 (€484,226)	$722,550 (€614,168)	−$152,872 (−€129,941)	$982,563 (€835,179)	$1,189,006 (€1,010,655)	−$206,443 (−€175,477)
**Total**	$627,559 (€533,425)	$760,382 (€646,325)	−$132,823 (−€112,900)	$1,232,003 (€1,047,203)	$1,477,277 (€1,255,685)	−$245,274 (−€208,483)	$1,826,468 (€1,552,498)	$2,119,229 (€1,801,345)	−$292,761 (−€248,847)

*Abbreviations*: *AE* adverse event, *ARTA* androgen receptor-targeted agent, *SSE* symptomatic skeletal event

Values correspond to a cohort of 100 patients for each treatment

## Discussion

The present cost–consequence analysis quantified the clinical and economic outcomes of using cabazitaxel versus a second ARTA as a 3 L treatment in patients with mCRPC previously treated with docetaxel and who progressed within 12 months while receiving an ARTA, from the US payers’ perspective. This study demonstrated that, at all assessed time points, cabazitaxel was associated with improved clinical outcomes (i.e., more patients achieving rPFS, PFS, and OS) and decreased HCRU costs associated with SSEs management and end-of-life care compared with that in a same-size cohort receiving a second ARTA.

Prostate cancer is associated with high HCRU [21]. Given that the occurrence of SSEs contributes significantly to the economic burden of patients with mCRPC, the reduction in costs associated with SSEs management is of particular interest [34]. Costs for the management of SSEs have been reported to constitute approximately 30% of the total claims for Medicare-enrolled men with prostate cancer in the US between the date experiencing the first SSE and the date of death [22]. In the CARD trial, cabazitaxel was associated with a 36% reduction in the risk of death due to any cause [19] and lower rates of SSEs despite the lower use of bone-targeted agent at baseline [25]. Importantly, our analysis estimated a 21% reduction in costs associated with SSEs management with the use of cabazitaxel as a 3 L treatment compared with a second ARTA at 18 months, primarily due to reduced hospitalization costs. In addition to the economic benefits, prevention or delay in SSEs improves the quality of life of patients with mCRPC [35–37].

In the present analysis, the estimate of AE-related costs was 10% higher for cabazitaxel at 18 months; however, this difference was offset by the cost savings related to SSE management and end-of-life care. Hospitalization costs were estimated to constitute approximately 90% (range: 87–91%) of the total costs for both cabazitaxel and a second ARTA at all assessed time points. Cabazitaxel was associated with a 17% decrease in hospitalization-related costs at 18 months. Overall, this cost–consequence analysis suggested that cabazitaxel as a 3 L treatment for patients with mCRPC, compared with a same-size cohort receiving a second ARTA, results in net savings with improvement in the time to disease progression. These results are in line with a previous budget impact analysis where a hypothetical increase (from 24 to 33%) in the use of cabazitaxel as a second-line treatment was estimated to result in cost savings of $86,136 (USD 2015) in patients with mCRPC previously treated with docetaxel compared with ARTAs [38].

Although a standard clinical treatment pattern has not been established for patients with mCRPC, under real-world conditions, abiraterone is increasingly being used as a second-line treatment owing to lower drug acquisition costs [39]. However, certain patients with mCRPC progress during treatment with an ARTA [40]. Thus, upon progression on an ARTA before or after docetaxel, clinicians can use either cabazitaxel or an alternative ARTA as a 3 L treatment. The available evidence from the CARD trial supports the use of cabazitaxel over a second ARTA in terms of both clinical outcomes (rPFS, PFS, and OS) and quality of life (pain response, pain progression, and SSEs) [19, 25]. The current analysis suggests that the use of cabazitaxel as a 3 L treatment for patients with mCRPC results in HCRU cost savings versus a second ARTA. Considering this evidence, cabazitaxel offers advantages in terms of improved clinical outcomes and lower healthcare costs versus a second ARTA (abiraterone or enzalutamide) for managing patients with mCRPC who were previously treated with docetaxel and had progressed while receiving an ARTA. Future research should include assessment of economic outcomes in parallel with clinical outcomes in primary comparative studies of various treatment options.

There are limitations associated with this analysis. These results reflect the outcomes expected for a patient population reflective of the CARD trial population; therefore, their generalizability to populations often not well represented in clinical trials, such as racial minorities and patients with poor performance or comorbidities, may be limited. Additionally, applicability of this data to other mCRPC patients (e.g., 2 L after 1 L ARTA [no prior chemotherapy], or prior treatment on ARTA more than 12 months) is also limited. The CARD trial evaluated the 25 mg/m^2^ dose of cabazitaxel, while 20 mg/m^2^ is routinely used in clinical practice in the US. However, no major difference in efficacy is expected between the two doses based on the PROSELICA study [41] and input from clinicians. Moreover, modelling the safety profile based on the 25 mg/m^2^ dose was considered a conservative approach for the analysis as this would bias the analysis to higher rates of complications and costs of treatment if bias was introduced. In real-world practice, patients treated with cabazitaxel are those who are generally assessed to have a better performance status and life expectancy than those who undergo a second ARTA, which may contribute to bias in the study results. This analysis is from the US payers’ and population healthcare decision makers’ perspective; therefore, indirect costs (e.g., caregiver burden, lost productivity, etc.) were not considered. Additionally, we considered only those Grade 3/4 AEs for which there is a need for hospitalization, which substantively affects the quality of life. The inclusion of Grades 1 and 2 AEs may minimally impact model projections. Lastly, for informing some model inputs (i.e., rates of hospitalization and LOS for most of the AEs and SSEs), oncology practicing clinicians’ feedback was utilized due to a lack of published literature.

## Conclusion

The use of cabazitaxel as a 3 L treatment after docetaxel and an ARTA in patients with mCRPC is estimated to result in clinical benefits (i.e., longer rPFS, PFS, and OS) and lower healthcare resource utilization (fewer hospitalization and ICU days), compared with a second ARTA.

## Supplementary Information

**Additional file 1.**

## Data Availability

The datasets used and/or analysed during the current study are available from the corresponding author on reasonable request.

## Abbreviations

AEs: Adverse events
ARTA: Androgen receptor-targeted agent
ASTI: Androgen-signaling-targeted inhibitor
CPT: Current Procedural Terminology
CRPC: Castration-resistant prostate cancer
HCRU: Healthcare resource utilization
CTCAE: Common Terminology Criteria for Adverse Events
ICU: Intensive care unit
KM: Kaplan–Meier
LOS: Length of stay
mCRPC: Metastatic castration-resistant prostate cancer
NCI: National Cancer Institute
OS: Overall survival
PFS: Progression-free survival
rPFS: Radiographic progression-free survival
SSE: Symptomatic skeletal event
3 L: Third-line
US: United States
USD: US dollar
