# Supplementary material for: An economic evaluation of cabazitaxel versus a second androgen receptor-targeted agent (ARTA) for patients with metastatic castration-resistant prostate cancer previously treated with docetaxel and an ARTA: the United States payer perspective
Source: BMC Health Serv Res. 2022 Jul 14;22:916. doi: 10.1186/s12913-022-08274-x (PMC9284907; doi:10.1186/s12913-022-08274-x)
Supplement: Supplementary file 1 — Additional file 1. [file 12913_2022_8274_MOESM1_ESM.docx]

**An economic evaluation of cabazitaxel versus a second androgen receptor-targeted agents (ARTA) for patients with metastatic castration-resistant prostate cancer previously treated with docetaxel and ARTA: The US payer perspective**

Table of Contents

[1. Supplementary Appendix 1: Kaplan–Meier estimates for rPFS, PFS, and OS from the CARD trial 2](#_Toc83892968)

[2. Supplementary Appendix 2: Rate of symptomatic skeletal events 3](#_Toc83892969)

[3. Supplementary Appendix 3: Total months of survival and number of SSEs at each time point 4](#_Toc83892970)

[4. Supplementary Appendix 4: Incidences of Grade 3/4 adverse events from the CARD trial 5](#_Toc83892971)

[5. Supplementary Appendix 5: Admission rates and length of stay 6](#_Toc83892972)

[6. Supplementary Appendix 6: Distribution of symptomatic skeletal events 7](#_Toc83892973)

[7. Supplementary Appendix 7: Proportion of total cycles administered and total AE-related costs at each time point 8](#_Toc83892974)

[8. Supplementary Appendix 8: Total US costs of hospitalization 9](#_Toc83892975)

# Supplementary Appendix 1: Kaplan–Meier estimates for rPFS, PFS, and OS from the CARD trial

For illustration: at 18 months, for a cohort of 100 patients receiving cabazitaxel, we estimated that 15 patients were radiographic-progression-free, five patients were progression-free, and 38 patients were alive.

| **Timepoint** | **rPFS** | | **PFS** | | **OS** | |
| --- | --- | --- | --- | --- | --- | --- |
|  | **Cabazitaxel​** | **ARTA​** | **Cabazitaxel​** | **ARTA​** | **Cabazitaxel​** | **ARTA​** |
| 6 months​ | 58%​ | 36%​ | 36% ​ | 16%​ | 86%​ | 81%​ |
| 12 months​ | 27%​ | 9%​ | 10%​ | 3%​ | 56%​ | 45%​ |
| 18 months​ | 15%​ | 6%​ | 5%​ | 3%​ | 38%​ | 21%​ |
| 24 months​ | 6%​ | 4%​ | 0% | 0% | 25%​ | 9%​ |

ARTA, androgen receptor-targeted agent; OS, overall survival; PFS, progression-free survival; rPFS, radiographic progression-free survival

# Supplementary Appendix 2: Rate of symptomatic skeletal events

|  | **Cabazitaxel** | **ARTA** |
| --- | --- | --- |
| Mean Follow-up (year)^a^ | 0.875 | 0.808 |
| Probability of Event^a^ | 0.186 | 0.278 |
| Annual Rate per Patient^b^ | 0.235 | 0.403 |
| Monthly Rate per Patient | 0.020 | 0.034 |

^a^Source: CARD trial[1]

^b^Calculated from follow-up time and probability of event based on the formula r = -(1/t)*LN(1-p), where r, rate; t, time; and p, probability; as described by Fleurence et al. (2007)[2]

ARTA, androgen receptor-targeted agent

# Supplementary Appendix 3: Total months of survival and number of SSEs at each time point

For illustration: total months of OS at 6 months = (number of survivors at 6 months x 6 months) + (number of deaths at 6 months x median OS at 6 months).

| **Time** | **Total months of survival** | | **Number of SSEs** | |
| --- | --- | --- | --- | --- |
|  | **Cabazitaxel** | **ARTA** | **Cabazitaxel** | **ARTA** |
| 6 months | 574 | 555 | 11 | 19 |
| 12 months | 1,034 | 934 | 20 | 31 |
| 18 months | 1,307 | 1,127 | 26 | 38 |
| 24 months | 1,485 | 1,210 | 29 | 41 |

Note: Values correspond to a cohort of 100 patients for each treatment.

ARTA, androgen receptor-targeted agent; OS, overall survival; SSE, symptomatic skeletal events

# Supplementary Appendix 4: Incidences of Grade 3/4 adverse events from the CARD trial

| **Grade 3/4 Adverse Event^a^** | **Cabazitaxel** | **ARTA** |
| --- | --- | --- |
| Asthenia or fatigue | 4% | 2% |
| Diarrhea | 3% | 0% |
| Infection | 8% | 7% |
| MSK pain or discomfort | 2% | 6% |
| Peripheral neuropathy | 3% | 0% |
| Renal disorder | 3% | 8% |
| Cardiac disorder | 1% | 5% |
| Febrile neutropenia | 3% | 0% |
| Anemia | 8% | 5% |
| Leukopenia | 32% | 2% |
| Neutropenia | 45% | 3% |
| Thrombocytopenia | 3% | 2% |
| Hyponatremia | 3% | 2% |

^a^Reported to occur in ≥3% of the patients in either treatment arm of the CARD trial[3]

ARTA, androgen receptor-targeted agent; MSK, musculoskeletal

#

# Supplementary Appendix 5: Admission rates and length of stay

| **Event** | **Hospitalization Rate (%)** | **Hospitalization Days** | **ICU Rate (%)** | **ICU Days** |
| --- | --- | --- | --- | --- |
| Pathological fracture | 100 | 5 | 0 | 0 |
| Spinal cord compression | 100 | 5 | 5 | 2 |
| Diarrhea | 100 | 2 | 0 | 0 |
| Infection | 100 | 4 | 20 | 2 |
| Renal disorder | 100 | 4 | 20 | 2 |
| Cardiac disorder | 100 | 4 | 20 | 2 |
| Febrile neutropenia | 100 | 4 | 20 | 2 |
| Anemia | 15 | 2 | 0 | 0 |
| Thrombocytopenia | 7.5 | 2 | 0 | 0 |
| Hyponatremia | 7.5 | 3 | 0 | 0 |
| End-of-life | 10 | 22 | 0 | 0 |

Note: Based on clinician input.

ICU, intensive care unit

# Supplementary Appendix 6: Distribution of symptomatic skeletal events

| **Type of SSE** | **Cabazitaxel^a^** | **ARTA^a^** |
| --- | --- | --- |
| Radiation to bone | 58% | 66% |
| Pathological fracture | 25% | 23% |
| Spinal cord compression | 17% | 11% |
| Surgery to bone | 0% | 0% |

^a^Obtained from CARD trial[1] and reweighted to add up to 100%.

ARTA, androgen receptor-targeted agent; SSE, symptomatic skeletal event

# Supplementary Appendix 7: Proportion of total cycles administered and total AE-related costs at each time point

| **Treatment** | **6 months** | **12 months** | **18 months** | **24 months** |
| --- | --- | --- | --- | --- |
| **% of total cycles administered**^a^ | | | | |
| Cabazitaxel | 81.9% | 96.5% | 99.7% | 100.0% |
| ARTA (pooled) | 78.2% | 90.6% | 96.9% | 98.8% |
| **Total AE-related costs**^b^ | | | | |
| Cabazitaxel | $226,750 (€192,738) | $267,456 (€227,338) | $276,198 (€234,768) | $277,018 (€235,465) |
| ARTA | $202,843 (€172,417) | $234,821 (€199,598) | $251,124 (€213,455) | $256,140 (€217,719) |

^a^Source: CARD trial[3]

^b^For a cohort of 100 patients for each treatment

AE, adverse event; ARTA, androgen receptor-targeted agent

# Supplementary Appendix 8: Total US costs of hospitalization

| **Time** | **Cabazitaxel** | **ARTA​** | **Difference** |
| --- | --- | --- | --- |
| 6 months​ | $548,407 (€466,146) | $666,932 (€566,892) | –$118,525 (–€100,746) |
| 12 months​ | $1,112,419 (€945,556) | $1,327,482 (€1,128,360) | –$215,063 (–€182,804) |
| 18 months​ | $1,442,870 (€1,226,440) | $1,728,394 (€1,469,135) | –$285,524 (–€242,695) |
| 24 months​ | $1,672,235 (€1,421,400) | $1,928,334 (€1,639,084) | –$256,099 (–€217,684) |

Note: Values correspond to a cohort of 100 patients for each treatment.

ARTA, androgen receptor-targeted agent

**References**

1. Fizazi K, Kramer G, Eymard JC, Sternberg CN, de Bono J, Castellano D, Tombal B, Wulfing C, Liontos M, Carles J *et al*: **Quality of life in patients with metastatic prostate cancer following treatment with cabazitaxel versus abiraterone or enzalutamide (CARD): an analysis of a randomised, multicentre, open-label, phase 4 study**. *Lancet Oncol* 2020, **21**(11):1513-1525.

2. Fleurence RL, Hollenbeak CS: **Rates and probabilities in economic modelling: transformation, translation and appropriate application**. *Pharmacoeconomics* 2007, **25**(1):3-6.

3. de Wit R, de Bono J, Sternberg CN, Fizazi K, Tombal B, Wulfing C, Kramer G, Eymard JC, Bamias A, Carles J *et al*: **Cabazitaxel versus Abiraterone or Enzalutamide in Metastatic Prostate Cancer**. *N Engl J Med* 2019, **381**(26):2506-2518.
